# Concomitant Use of Dietary Supplements and Medicines Among Preschool and School-Aged Children in Japan

**DOI:** 10.3390/nu11122960

**Published:** 2019-12-04

**Authors:** Etsuko Kobayashi, Yoko Sato, Chiharu Nishijima, Tsuyoshi Chiba

**Affiliations:** Department of Food Function and Labeling, National Institute of Health and Nutrition, National Institutes of Biomedical Innovation, Health and Nutrition, 1-23-1 Toyama, Shinjuku-ku, Tokyo 162-8636, Japan; e-kbyshi@nibiohn.go.jp (E.K.); satoyoko@nibiohn.go.jp (Y.S.);

**Keywords:** dietary supplements, children, mothers, adverse event, internet survey

## Abstract

Dietary supplement use is widespread amongst the general population including in children and adolescents. The ingredients in dietary supplements can interact with medicines when patients take them concomitantly. However, the prevalence of the concomitant use of dietary supplements and medicines in Japan among children remains unclear. To clarify this issue, a nationwide internet survey was administered to 55,038 mothers (25 to 60 years old) of preschool- or school-aged children in Japan. Among them, 7.6% currently provide dietary supplements and 3.2% concomitantly provide dietary supplements and prescription or over-the-counter medicines to their children. The prevalence of concomitant use increased with the children’s grade. Among 1057 mothers with 1154 children who were concomitantly using dietary supplements and medicines, 69.1% provided dietary supplements without physician consultation because they considered dietary supplements as only foods and therefore safe. Although the purpose of the use and types of dietary supplement differed between boys and girls, the most popular product was probiotics in both boys and girls. Among concomitant users, 8.3% of mothers gave dietary supplements for treatment of diseases and 4.9% mothers recognized the adverse events of dietary supplements in their child. The findings of this study suggest that mothers’ knowledge about the risk of using dietary supplement with medicines is insufficient. Parental education about the safety of dietary supplements and potential risk of drug–supplement interaction is needed.

## 1. Introduction

The use of dietary supplements has been increasing worldwide. In the USA, over half of adults use dietary supplements [1,2]. Vitamins and minerals are the popular ingredients, and the common reasons for dietary supplement use are to improve or maintain health [3]. In Japan, about 30% of healthy people use dietary supplements [4]. In addition to vitamins and minerals, people also use non-vitamin and non-mineral supplements such as fish oil, probiotics, and herbal products [5]. Dietary supplements are not regulated by law in Japan and their safety and effectiveness is not strictly evaluated, as medicines are. However, sometimes dietary supplements are used among patients to treat their diseases [5,6,7]. As various ingredients are concentrated in dietary supplements into tablets or capsules, the potential adverse effects are cause for concern. Despite the adverse events associated with dietary supplements being usually mild or moderate [8], severe cases such as liver injury have been reported [9]. The ingredients of dietary supplements, especially herbs, can interact with medicines, via interference to absorption, bioavailability, or metabolism of medicines [10,11]. In addition, it is also reviewed that the phytopharmaceuticals in dietary supplements increase or decrease cytochrome P-450 (CYP) activities in vivo studies [12]. If patients take dietary supplements and medicines concomitantly, efficacy of medicines may increase or decrease depending on an interaction manner. Although potential drug-supplement interaction is concerning, most of dietary supplement users do not consult health professionals about concomitant use of dietary supplement and medicines [4].

Dietary supplement use is widespread in the general population, including in children and adolescents. In the USA, about one-third of children was used dietary supplements [13,14] and dietary supplement use is expanding even among infants and toddlers [15]. The prevalence of dietary supplement use under the age of 18 years was also reported from Europe [16,17], and Asian regions [18,19]. In Japan, the prevalence of dietary supplement use was reported at 8.0% in 2009 [20] and 6.1% in 2016 [21] among preschool children (daily or occasional use) as well as 16.4% in 2018 among elementary to high school students [22]. Maintaining health is a common reason for dietary supplement use among children. However, in our previous study, some mothers provided dietary supplement to their child for treatment of diseases. Some of them believed that dietary supplements can treat diseases or can be used concomitantly with drugs [22]. It is also reported that the prevalence of use of vitamin/mineral [23,24] or functional dietary supplement [24] was higher in children with chronic diseases than healthy counterparts, and that dietary supplement use was more common in children who used medicines than non-users [25]. However, previous study showed that probable or possible adverse events due to natural health herb products and drug interaction were occurred among pediatric patients with attention-deficit/hyperactivity disorder [26].

Previously, we reported the prevalence of dietary supplement use in children [22]. The prevalence was increased with their age, and the purpose and products they used were different among sex or age. We also reported that the prevalence of concomitant use of dietary supplements and medicines among adult patients in Japan was 37% in ambulatory patients and 18% in admitted patients, and 3.3% of them experienced adverse events [4]. However, the prevalence of the concomitant use of dietary supplements and medicines among children in Japan remains unclear. Furthermore, it is also unclear whether adverse events associated to concomitant use of dietary supplements and medicines occur in children. To clarify this issue, we conducted a nationwide online survey about the concomitant use of dietary supplements and medicines in children in Japan.

## 2. Materials and Methods

### 2.1. Internet Survey and Participants

An Internet-based survey consisting of with two phases (preliminary survey and targeted survey) was conducted by Cross Marketing Inc. (Tokyo, Japan), from 26 September to 23 October 2018. We sent an invitation by mail to 447,101 mothers aged from 25 to 60 years old who had preschool- or school-aged children to participate in the preliminary survey. Of these, 55,038 mothers responded (response rate: 12.3%). Mothers whose children were concomitantly using dietary supplements and medicines were selected from participants of the preliminary survey and were asked to answer the targeted survey questionnaire. In the targeted survey, mothers who had multiple target-aged children were asked to answer about up to three of their children from the youngest child. In total, 1057 mothers with 1154 children participated in the targeted survey. Children were classified according to sex and age or school grade group: One to three years, four to six years (preschool), elementary school (ES) (first to third grade) and ES (fourth to sixth grade), junior high school (JHS), and high school (HS). This study was conducted with the approval of the Research Ethics Committee of the National Institutes of Biomedical Innovation, Health and Nutrition (No. 133, approved on 12 June 2018), and in accordance with the Declaration of Helsinki.

### 2.2. Questionnaires for the Preliminary and Targeted Surveys

In the preliminary survey, mothers answered about their children’s dietary supplement use (“currently using”, “previously used”, or “never used”), prescription, and/or over-the-counter (OTC) medicine use and concomitant use of dietary supplements and medicines in addition to providing demographic information (residence area, age, and school grade of their child).

In the targeted survey, the questionnaire included demographic characteristics of children (sex and age groups), the purpose of the use of dietary supplements, the types of dietary supplements, and adverse event experiences. The questionnaire also asked whether mothers informed their children’s physicians or pharmacists about dietary supplement use, and, if not, the reasons why.

In this survey, we defined dietary supplements as foods that were in the form of capsules, tablets, and powders and that were considered to have beneficial effects on the children’s health. The mothers were informed of the definition of dietary supplement at the beginning of the questionnaire.

### 2.3. Statistical Analysis

Differences in the purposes of dietary supplement use among age groups were determined using chi-square test or Fisher’s exact test. Tests for trends in prevalence of dietary supplement use, medicine use, and concomitant use across age groups were performed using Stata nptrend (extension of the Wilcoxon rank-sum test). All statistical analyses were performed using Stata/IC 15 (Light Stone, Tokyo, Japan) and a *p*-value of <0.05 was considered statistically significant.

## 3. Results

### 3.1. Preliminary Survey

In total, 55,038 mothers completed the preliminary survey (mean age = 36.5 years). Age groups and grade of the children in the preliminary survey were as follows: One to three years (*n* = 17,361), four to six years (*n* = 9044), ES (first to third) grade (*n* = 8206), ES (fourth to sixth) grade (*n* = 7333), JHS (*n* = 7245), and HS (*n* = 5849).

The prevalence of dietary supplements, medicines, and concomitant use among children in the preliminary survey is outlined in Table 1. Among all children, 7.6% were currently using dietary supplements, 22.1% regularly took medicine, and 3.2% were concomitantly using dietary supplements and medicine. The prevalence of medicine increased by 25.0% in children four to six years old and then decreased to 22.2% in HS-aged children. However, the prevalence of dietary supplement use and concomitant use increased with children’s age (*p* for trend <0.01). The prevalence of dietary supplement use was higher among children who took medicines regularly (14.6%; 1,769/12,144) than those who did not use medicines (5.6%; 2,413/42,894).

### 3.2. Characteristics of the Targeted Survey

The characteristics of the children whose mothers completed the targeted survey (*n* = 1154) are provided in Table 2.

### 3.3. Purpose of Dietary Supplement Use and Types of Dietary Supplements

The purpose of dietary supplement use is shown in Table 3. “Supplementation of nutrition” was the most frequent answer in mothers of both boys and girls, followed by “maintenance of health/prevention of disease” and “improvements to health”. More boys’ mothers provided dietary supplements to “enhance growth” and this increased with age until ES fourth to sixth, and decreased in HS. Girls’ mothers were likely to provide products for the purpose of “beauty/weight loss” and this increased in HS. “Beauty/weight loss” (including skin repair) was also high in HS boys (11.4%). Overall, 8.3% of mothers (8.0% in boys, 8.7% in girls) provided dietary supplements for treatment of diseases.

The types of dietary supplements used are listed in Table 4. Regarding vitamin/mineral supplements, multi-vitamins were more popular than individual vitamins, whereas multi-minerals were less likely to use than individual minerals. Vitamin C was the most popular individual vitamin supplement (7.1%). Individual vitamin was more popular among HS than younger age groups in girls whereas no differences was found between age groups in boys. In boys, the most popular mineral supplement was calcium (7.9%). The mineral most used by girls was iron (8.9%) and the prevalence of individual mineral use was significantly high in JHS. Among non-vitamin and non-mineral supplements, probiotics were the most popular dietary supplement in both boys and girls (18.2% in boys, 18.5% in girls). The prevalence of probiotics was higher among young age groups in both boys (one to three years, four to six years, and ES first to third) and all age groups in girls compared to other non-vitamin/non-mineral supplements. Growth-promoting products were more popular in boys (15.1%) than girls (5.1%). Overall, 5.3% of children used botanical supplements. In this category, most of children used Aojiru products, and individual herbs were rarely used.

### 3.4. Types of Medicines

The types of medicines concomitantly used with dietary supplements are listed in Table 5. Medicines for allergic diseases such as rhinitis, eczema, and asthma were commonly used, and medicines for gastrointestinal diseases and psychiatric disorders followed for both boys and girls. The prevalence of the concomitant use of individual types of dietary supplements and medicines is provided in Table 6. Probiotics were more popular in children who were taking medicine for allergic diseases. Approximately half of the children who were taking prescription or OTC medicine concomitantly used vitamin/mineral dietary supplements.

We also analyzed the difference of the concomitant use of individual types of dietary supplements among different sex and ages in each medicine (Supplementary Tables S1–S8). In this analysis, we could not find the difference between boys and girls or among ages in each medicine. However, most of vitamin/mineral medicine users took vitamin/mineral dietary supplements. (Supplementary Table S8)

### 3.5. Consultation About Dietary Supplement Use with Physicians or Pharmacists

Only 30.8% of mothers consulted with their children’s physicians or pharmacists about dietary supplement use. We inquired as to the reason for the lack of consultation from 798 mothers who had not consulted with physicians or pharmacists. For this question, 45.1% of mothers answered that dietary supplements were just foods and 25.8% of mothers answered that dietary supplement did not affect their medication. In addition, 19.3% of mothers answered that physicians or pharmacists have never asked them about dietary supplement use.

### 3.6. Adverse Event Experience

Overall, 4.9% of mothers reported adverse events after using dietary supplements in their children (Table 7). The prevalence of adverse event was slightly higher in girls (5.1%) compared to boys (4.6%), and in younger compared to older children. Among them, the most frequently reported symptom was diarrhea (44.6%), followed by constipation (19.6%), nausea/vomiting (16.1%), and stomachache (14.3%). Adverse events were more frequent in children who received dietary supplements to treat disease (16.7%; 16/96) than in children who received dietary supplements for other purposes (3.8%; 40/1,058).

## 4. Discussion

In the present study, the prevalence of dietary supplement use was increased with children’s age from 3.5% in one to three years to 14.4% in HS. “Supplementation of nutrition” was the most frequent purpose of dietary supplement use, and “enhance growth” was high in boys, and “beauty/weight loss” was high in girls. This trend is consistent with our previous report [22]. However, the prevalence in adolescents was still lower than in the USA (32%) [13] or European countries (16.4–69.0%) [16,17,19]. New finding in this study is that the ratio of concomitant use of dietary supplement and medicine was also increased with children’s age from 1.4% in one to three years to 6.0% in HS. Medicines for rhinitis, eczema, asthma, and probiotics as non-vitamin/non-mineral dietary supplements were popular in both boys and girls. It might be caused because mothers believed that probiotics were good for allergy disease via improvement of immune system.

Mothers of children who were administered medicine were more likely to give them dietary supplements than mothers whose children did not take medicines in this study. Similarly, it was reported that dietary supplement use was more common in those who used prescription medications and among those who had a diagnosis of chronic headaches among adolescents in the USA [25]. In addition, the presence of chronic disease is associated with dietary supplement use among children in South Korea [24] and Poland [23]. These situation is also reported in adults. One-fourth of vitamin/mineral supplement users were using supplements to support disease treatment in Germany [27]. Herbal product use was higher among patients taking prescription or OTC medicines in the USA [25,28]. Another study reported that the prevalence of dietary supplement was greater in people with health problems compared to healthy subjects [29]. In Japan, a retrospective study also showed health foods/supplements use was higher in medicated patients than non-medicated patients [30]. These reports, including our present study, showed that the prevalence of dietary supplement use is greater in people who have some problem in their health compared to health subjects, and it occurred in not only adult, but also children. However, the ingredients in dietary supplements can interact with medicines and some patients may be concomitantly using potential harmful combination of dietary supplement and medicine [26]. Mothers of children who take medicines for chronic health problems should be informed about the risks of concomitant use of dietary supplements and medicine.

The rates of patients’ disclosure of dietary supplements or other complementary or alternative medicine use to health professionals vary. A meta-analysis showed that the disclosure rate of complementary medicine was 33% [31]. In the present study, less than one-third of mothers of concomitant users had consulted with health professionals about their children’s dietary supplement use. Most of mothers who did not consult with a health professional believed that dietary supplements are safe and do not interfere with medications. This recognition was almost the same in a survey of adults who used dietary supplements alone [5]. However, children are more affected not only by medicines, but also by dietary supplements. To avoid adverse events due to drug–supplement interactions, parental education about safety of dietary supplementation is important.

Among mothers of concomitant users, 32.8% provided vitamin/mineral supplements. Vitamins and minerals were also used as medicines. The prevalence of vitamin/mineral supplement use was higher among prescription or OTC vitamin/mineral medicines users (52.7%; 69/131) than non-users (30.3%; 310/1,023). However, because dietary supplements and medicines appear similar, some mothers may confuse them. In this study, some mothers inserted the name of a dietary supplement as a medicine name and vice versa. Some of them provided the same product name as both a dietary supplement and a medicine. Therefore, the prevalence rate of the concomitant use of vitamin/mineral dietary supplement and vitamin/mineral medicines may be overestimated.

In addition to vitamin/mineral supplements, mothers provided various types of dietary supplements to their children and the trend differed from that identified in our previous survey. In the past study, protein/amino acids was the most popular non-vitamin non-mineral supplement among boys, used by 33% of HS boys [22]. In the present study, although prevalence of protein/amino acid products was slightly higher among boys than girls, the rate ranged from 0% (four to six years) to 11.4% (HS). Instead, probiotics were the most popular non-vitamin, non-mineral supplement in all age and sex groups, except for HS boys. The prevalence was high particularly in young children (one to three years of age, 22.1%; four to six years of age, 33.8%). Probiotics are believed to improve immune system and is a popular dietary supplement among those who are ill within the population. However, a cohort study reported that probiotics usage is associated with health problems such as eczema and gastrointestinal tract problems in young children [32]. A review reported that having a health problem was one of predictors of children’s probiotics or dietary supplement use [33]. In the present study, many of children took medicines for allergic diseases such as allergic rhinitis (40.0%), eczema (36.0%), and asthma (31.8%). The prevalence of probiotics use was higher among children who used medicines for allergic rhinitis, asthma, or eczema than non-users (23.6% versus 14.9%, 22.2% versus 16.3%, and 23.4% versus 16.0%, respectively; all *p* < 0.05). Therefore, mothers may provide probiotic products for their children to treat mild chronic conditions. Most of the mothers gave their children probiotics for “improvements in health” (71.7%). A systematic review of randomized controlled trials showed that probiotics have a beneficial effect on rhinitis patients’ quality of life scores but had no effect on symptom scores [34]. Another review reported that probiotics significantly affected the symptoms of allergic rhinitis, but high heterogeneity was observed partly due to differences of strains among studies [35]. Probiotics are probably safe in general populations; however, some probiotic products contain hidden milk or egg protein and can cause anaphylaxis among children with food allergies [36,37]. Mothers should provide probiotic products for children with allergies with caution and should consult with a doctor before providing supplementation.

Overall, some mothers realized that adverse events could possibly be related to dietary supplement use among their children. In line with our previous study in healthy children [22], most events were gastrointestinal symptoms, such as diarrhea and constipation, and these events are less likely to be caused by drug–supplement interaction effects. Although the causal relationship between supplement use and adverse events was not clear, adverse events were more frequent when children were provided dietary supplements to treat disease (16.7%) compared to other purposes (3.8%). This trend is similar to that found in a previous study among an adult population [5]. At this time, the reason for this finding is unclear, but children who take medicines already have some health problems, so dietary supplements may easily cause adverse events.

The present study has several limitations. First, this was an Internet survey and we recruited participants from monitors of a research company. Therefore, the participants may not fully represent the general population. Second, some consumers in Japan confused dietary supplements and medicines. Dietary supplement has no clear definition in the law in Japan. So, some participants confused medicines as dietary supplements, even though we presented the definition of dietary supplement before the questionnaire. Adverse events reported in this study were self-reported by mothers, and we cannot assess dose, duration, and timing of use of dietary supplements and medicines. In addition, we also did not ask children’s anamnesis which might affect the prevalence of adverse events associate to dietary supplement use. Therefore, we were not able to infer any causal relationship. To clarify the causal relationship of adverse events, the questionnaire also needs to be reexamined to make it better to collect detail information more effectively in the further study. However, our results reveal the necessity of parental education about dietary supplement at the moment.

## 5. Conclusions

In the present study, we conducted a nationwide survey and revealed the prevalence of concomitant use of dietary supplements and medicine among children in Japan. Some mothers provided dietary supplements and medicines to their children concomitantly. The rate of concomitant users increased with children’s age (from 1.4% in one to three years to 6.0% in HS). Overall, 8.3% of mothers who gave dietary supplements for treatment of diseases. Among concomitant users, 4.9% of mothers noticed adverse events associated with dietary supplement use, and the ratio of adverse events might be higher among children who received dietary supplements to treat their diseases. In this survey, we could not define the causal relationship of these adverse events, and further study is needed. However, to prevent adverse events caused by drug–supplement interactions, education for parents about the potential risk of dietary supplement use in ill children is needed.

## Figures and Tables

**Table 1:** The prevalence of dietary supplement use, medicine use, and concomitant use of dietary supplements and medicines in children in the preliminary survey (%).

Age/Grade ^1^	*N*	Dietary Supplements		Medicine		Concomitant Use	
**All**	55,038	7.6		22.1		3.2	
			*p* for trend		*p* for trend		*p* for trend
1–3 years	17,361	3.5	<0.01	18.6	<0.01	1.4	<0.01
4–6 years	9044	6.2		25.0		2.9	
ES (1st–3rd)	8206	7.9		24.5		3.5	
ES (4th–6th)	7333	8.8		24.0		3.6	
JHS	7245	12.0		21.9		5.0	
HS	5849	14.4		22.2		6.0	

^1^ ES, elementary school; JHS, junior high school; and HS, high school. Tests for trend among age groups were performed using nptrend command (Stata, Light Stone, Tokyo, Japan).

**Table 2:** Characteristics of the children whose mothers completed the targeted survey.

Variable	*N*	%
**All**	1154	
**Sex**		
Boys	647	56.1
Girls	507	43.9
**Age/Grade**		
1–3 years	95	8.2
4–6 years	157	13.6
ES (1st–3rd)	193	16.7
ES (4th–6th)	199	17.2
JHS	262	22.7
HS	248	21.5
**Residential area**		
Hokkaido	41	3.6
Tohoku	88	7.6
Kanto	440	38.1
Chubu	202	17.5
Kinki	199	17.2
Chugoku	55	4.8
Shikoku	26	2.3
Kyusyu/Okinawa	103	8.9

**Table 3:** What is the purpose of dietary supplement use? (%).

	Boys (*n* = 647)								Girls (*n* = 507)								*p*-Value ^2^
	*n*	1–3 Years (57)	4–6 Years (92)	ES 1st–3rd (118)	ES 4th–6th (126)	JHS (131)	HS (123)	*p*-Value ^1^	*n*	1–3 Years (38)	4–6 Years (65)	ES 1st–3rd (75)	ES 4th–6th (73)	JHS (131)	HS (125)	*p*-Value ^1^	
Supplementation of nutrition	331	61.4	51.1	52.5	51.6	53.4	42.3	0.25	251	42.1	61.5	58.7	49.3	45.0	44.8	0.10	0.58
Maintenance of health/prevention of disease	286	40.4	44.6	48.3	42.1	46.6	41.5	0.84	237	60.5	40.0	46.7	49.3	48.9	42.4	0.36	0.39
Improvements to health	203	31.6	32.6	30.5	33.3	30.5	30.1	0.99	176	31.6	41.5	34.7	30.1	40.5	28.8	0.31	0.23
Enhance growth	140	17.5	15.2	17.8	31.0	28.2	15.4	0.01	49	7.9	9.2	9.3	15.1	11.5	5.6	0.36	<0.01
Treatment of disease	52	10.5	7.6	9.3	9.5	6.1	6.5	0.82	44	15.8	10.8	6.7	6.8	9.9	6.4	0.47	0.70
Beauty/weight loss	23	1.8	2.2	2.5	1.6	0.8	11.4	<0.01	67	5.3	4.6	6.7	5.5	16.0	25.6	<0.01	<0.01
Enhance athletic performance	51	3.5	7.6	6.8	7.1	8.4	11.4	0.56	27	2.6	9.2	6.7	8.2	3.1	4.0	0.32	0.09
Enhance academic performance	41	3.5	9.8	9.3	7.1	3.1	4.9	0.20	30	2.6	6.2	6.7	5.5	6.1	6.4	0.99	0.77
No reason	11	-	4.3	3.4	0.8	1.5	-	-	11	2.6	4.6	4.0	2.7	-	1.6	-	0.56
Others	17	-	1.1	1.7	3.2	3.1	4.9	-	12	2.6	3.1	2.7	1.4	2.3	2.4	-	0.78

^1^ Statistical analyses were conducted among age groups using chi-square test or Fisher’s exact test. ^2^ Statistical analyses were conducted among boys and girls using chi-square test. Note: Multiple answers.

**Table 4:** What kind of dietary supplements are you giving your child? (%).

	Boys (*n* = 647)								Girls (*n* = 507)								*p*-Value ^2^
	*n*	1–3 Years (57)	4–6 Years (92)	ES 1st–3rd (118)	ES 4th–6th (126)	JHS (131)	HS (123)	*p*-Value ^1^	*n*	1–3 Years (38)	4–6 Years (65)	ES 1st–3rd (75)	ES 4th–6th (73)	JHS (131)	HS (125)	*p*-Value ^1^	
Vitamin/Mineral																	
Multi-vitamins and minerals	35	3.5	3.3	6.8	4.0	6.9	6.5	0.70	24	5.3	-	2.7	6.8	6.1	5.6	-	0.61
Multi-vitamins	57	12.3	13.0	10.2	4.8	6.9	8.9	0.27	58	7.9	9.2	14.7	6.8	11.5	14.4	0.53	0.14
Individual vitamin	49	3.5	4.3	6.8	5.6	10.7	11.4	0.16	61	2.6	6.2	14.7	6.8	12.2	19.2	0.02	0.01
Multi-minerals	5	-	-	0.8	0.8	0.8	1.6	-	5	2.6	1.5	-	2.7	-	0.8	-	-
Individual mineral	67	12.3	5.4	10.2	8.7	12.2	13.0	0.49	64	7.9	7.7	4.0	16.4	17.6	14.4	0.04	0.23
Any type	193	31.6	26.1	30.5	22.2	31.3	37.4	0.17	186	26.3	21.5	34.7	34.2	42.7	44.0	0.02	0.01
Non-Vitamin, Non-Mineral																	
Probiotics	118	15.8	35.9	19.5	19.0	14.5	8.1	<0.01	94	31.6	30.8	24.0	17.8	16.8	7.2	<0.01	0.90
Growth-promoting	98	12.3	8.7	16.1	20.6	19.1	10.6	0.08	26	5.3	6.2	10.7	9.6	2.3	1.6	-	<0.01
Protein/Amino acid	38	1.8	-	0.8	8.7	8.4	11.4	<0.01	14	2.6	3.1	5.3	5.5	0.8	1.6	-	0.01
Academic enhancement	21	3.5	4.3	2.5	3.2	6.1	-	-	14	2.6	6.2	5.3	1.4	2.3	0.8	-	0.63
Botanical nutrients/Aojiru ^1^	39	3.5	-	5.9	7.9	6.9	8.9	0.10	22	7.9	1.5	2.7	4.1	5.3	4.8	-	0.20
*n*-3 PUFA	39	8.8	3.3	5.9	9.5	3.8	5.7	0.32	21	5.3	4.6	2.7	4.1	1.5	7.2	-	0.15
Cod liver oil	25	8.8	6.5	5.1	4.8	1.5	-	-	17	2.6	12.3	6.7	1.4	1.5	-	-	0.65
Eye care ^2^	19	-	-	2.5	3.2	4.6	4.9	-	16	-	-	1.3	4.1	6.9	2.4	-	0.83
Skin repairing	5	-	-	0.8	-	2.3	0.8	-	7	-	-	-	-	0.8	4.8	-	0.31
Weight loss	0	-	-	-	-	-	-	-	3	-	-	-	-	0.8	1.6	-	-
Others	96	12.3	15.2	18.6	12.7	16.0	13.0	-	99	15.4	10.7	16.4	19.8	31.2	15.6	-	-

^1^ Statistical analyses were conducted among age groups using chi-square test. ^2^ Statistical analyses were conducted among boys and girls using chi-square test. PUFA: Polyunsaturated fatty acid. ^1^ A powdered drink mix made from green leafy vegetables such as young leaves of *Angelica keiskei* (Miq.) Koidz and Barley, and *Brassica oleracea* L. var. *acephala* DC. ^2^ Products containing anthocyanin, lutein, and zeaxanthin. Note: Multiple answers.

**Table 5:** What kind of medicines are you giving your child? (%).

	Boys (*n* = 647)								Girls (*n* = 507)								*p*-Value ^2^
	*n*	1–3 Years (57)	4–6 Years (92)	ES 1st–3rd (118)	ES 4th–6th (126)	JHS (131)	HS (123)	*p*-Value ^1^	*n*	1–3 Years (38)	4–6 Years (65)	ES 1st–3rd (75)	ES 4th–6th (73)	JHS (131)	HS (125)	*p*-Value ^1^	
Rhinitis	282	38.6	33.7	42.4	54.0	46.6	40.7	0.06	180	18.4	33.8	40.0	49.3	35.1	31.2	0.03	<0.01
Eczema	257	35.1	30.4	39.8	51.6	38.2	38.2	0.04	158	21.1	29.2	41.3	41.1	29.0	25.6	0.05	<0.01
Asthma	226	47.4	42.4	38.1	38.9	26.7	25.2	<0.01	141	31.6	49.2	33.3	28.8	26.7	12.8	<0.01	0.01
Gastrointestinal diseases	58	21.1	10.9	5.1	7.1	7.6	8.9	0.02	52	13.2	9.2	14.7	8.2	7.6	11.2	0.63	0.46
Psychiatric disorder	61	-	1.1	12.7	17.5	9.9	8.1	<0.01	46	2.6	3.1	10.7	6.8	7.6	16.0	-	0.84
Antibiotics	16	1.8	1.1	1.7	1.6	2.3	5.7	-	18	10.5	4.6	4.0	6.8	1.5	0.8	-	0.28
Painkiller	11	-	1.1	1.7	-	3.1	3.3	-	18	5.3	1.5	1.3	1.4	3.1	7.2	-	0.05
Vitamin/mineral	60	1.8	7.6	5.9	6.3	12.2	17.1	<0.01	71	10.5	7.7	10.7	8.2	19.8	17.6	0.07	0.01

^1^ Statistical analyses were conducted among age groups using chi-square test. ^2^ Statistical analyses were conducted among boys and girls using chi-square test. Note: Multiple Answers.

**Table 6:** Types of medicines concomitantly used with individual dietary supplements. (%).

	Vitamin/Mineral (379)	Probiotics (212)	Growth-Promoting (124)	Protein/Amino Acid (52)	Academic Enhancement (35)	Botanical Nutrients/Aojiru ^1^ (61)	*n*-3 PUFA (60)	Cod Liver Oil (42)	Eye Care ^2^ (35)	Skin Repairing (12)	Weight Loss (3)
Rhinitis (462)	31.6	23.6	11.9	3.7	3.0	6.5	5.8	3.7	3.0	1.3	0.2
Eczema (415)	32.3	22.2	12.0	3.4	2.9	6.3	5.3	3.6	2.4	1.4	0.2
Asthma (367)	30.8	23.4	12.3	5.4	4.6	4.4	4.1	6.8	2.2	1.1	-
Gastrointestinal diseases (110)	38.2	14.5	3.6	4.5	-	6.4	6.4	2.7	3.6	-	-
Psychiatric disorder (107)	36.4	14.0	10.3	3.7	4.7	8.4	9.3	-	2.8	1.9	-
Antibiotics (34)	20.6	23.5	5.9	5.9	-	5.9	2.9	8.8	2.9	-	-
Painkiller (29)	44.8	10.3	-	13.8	6.9	-	-	-	3.4	6.9	-
Vitamin/mineral (131)	52.7	4.6	5.3	3.1	1.5	3.1	3.1	3.1	3.8	-	-

^1^ A powdered drink mix made from green leafy vegetables such as young leaves of *Angelica keiskei* (Miq.) Koidz and Barley, and *Brassica oleracea* L. var. *acephala* DC. ^2^ Products containing anthocyanin, lutein, and zeaxanthin. Note: Multiple answer.

**Table 7:** Has your child ever experienced adverse events due to dietary supplement use? If yes, what symptom(s) did your child experience? (%).

	All (1154)	Boys (647)	Girls (507)	1–3 Years (95)	4–6 Years (157)	ES 1st–3rd (193)	ES 4th–6th (199)	JHS (262)	HS (248)
Yes	4.9	4.6	5.1	6.3	7.6	4.7	2.0	5.3	4.4
Symptom ^1^									
Diarrhea	44.6	53.3	34.6	66.7	50.0	44.4	25.0	50.0	27.3
Constipation	19.6	23.3	15.4	16.7	16.7	33.3	-	28.6	9.1
Nausea and vomiting	16.1	16.7	15.4	-	-	33.3	-	14.3	36.4
Headache	10.7	13.3	7.7	-	25.0	11.1	25.0	7.1	-
Stomachache	14.3	13.3	15.4	-	-	33.3	-	21.4	18.2
Eczema and itching	12.5	10.0	15.4	16.7	-	11.1	25.0	14.3	18.2
Influence the effectiveness of medicines	7.1	6.7	7.7	16.7	16.7	11.1	-	-	-
Fatigue	3.6	3.3	3.8	-	-	22.2	-	-	-
Others	8.9	3.3	15.4	-	-	-	25.0	21.4	9.1

^1^ All (*n* = 56), boys (*n* = 30), girls (*n* = 26), 1–3 years (*n* = 6), 4–6 years (*n* = 12), ES 1st–3rd grade (*n* = 9), ES 4th–6th grade (*n* = 4), JHS (*n* = 14), and HS (*n* = 11). Note: Multiple answers.

## Supplementary Materials

The following are available online at https://www.mdpi.com/2072-6643/11/12/2960/s1, Table S1: Types of dietary supplements concomitantly used with rhinitis medicines, Table S2: Types of dietary supplements concomitantly used with eczema medicines, Table S3: Types of dietary supplements concomitantly used with asthma medicines, Table S4: Types of dietary supplements concomitantly used with gastrointestinal diseases medicines, Table S5: Types of dietary supplements concomitantly used with psychiatric disorder medicines, Table S6: Types of dietary supplements concomitantly used with antibiotics, Table S7: Types of dietary supplements concomitantly used with pain killer, Table S8: Types of dietary supplements concomitantly used with vitamin/mineral medicines.
