# Supplementary material for: Concomitant Use of Dietary Supplements and Medicines Among Preschool and School-Aged Children in Japan
Source: Nutrients. 2019 Dec 4;11(12):2960. doi: 10.3390/nu11122960 (PMC6950417; doi:10.3390/nu11122960)
Supplement: Supplementary file 1 [file nutrients-11-02960-s001.pdf]

nutrients

MDPI

Supplementary tables

Table S-1. Types of dietary supplements concomitantly used with rhinitis medicines. (%)

	Boys (n=282)							Girls (n=180)						
	N	1-3 years (22)	4-6 years (31)	ES 1st-3rd (50)	ES 4th-6th (68)	JHS (61)	HS (50)	N	1-3 years (7)	4-6 years (22)	ES 1st-3rd (30)	ES 4th-6th (36)	JHS (46)	HS (39)
Vitamin/Mineral	76	27.3	19.4	28.0	19.1	24.6	44.0	70	57.1	18.2	26.7	44.4	43.5	46.2
Probiotics	65	22.7	45.2	18.0	26.5	19.7	14.0	44	-	36.4	30.0	25.0	28.3	12.8
Growth-promoting	47	9.1	12.9	16.0	19.1	21.3	14.0	8	-	9.1	13.3	5.6	-	-
Protein/amino acid	15	-	-	-	5.9	9.8	10.0	2	-	4.5	-	2.8	-	-
Academic enhancement	8	-	3.2	2.0	2.9	6.6	-	6	-	9.1	6.7	2.8	2.2	-
Skin repairing	3	-	-	-	-	3.3	2.0	3	-	-	-	-	2.2	5.1
Weight loss	0	-	-	-	-	-	-	1	-	-	-	-	2.2	-
Botanical nutrients/ Aojiru[1]	18	4.5	-	6.0	10.3	8.2	4.0	12	28.6	4.5	-	8.3	6.5	7.7
n-3 PUFA	17	9.1	3.2	8.0	7.4	3.3	6.0	10	-	-	6.7	8.3	-	12.8
Cod liver oil	10	9.1	3.2	6.0	5.9	-	-	7	14.3	18.2	3.3	2.8	-	-
Eye care[2]	8	-	-	2.0	1.5	3.3	8.0	6	-	-	-	2.8	8.7	2.6

[1]A powdered drink mix made from green leafy vegetables such as young leaves of *Angelica keiskei* (Miq.) Koidz and Barley, and *Brassica oleracea* L. var. *acephala* DC. [2]Products containing anthocyanin, lutein, and zeaxanthin. Note: Multiple answers.

Table S-2. Types of dietary supplements concomitantly used with eczema medicines. (%)

	Boys (n=257)							Girls (n=158)						
	N	1-3 years (20)	4-6 years (28)	ES 1st-3rd (47)	ES 4th-6th (65)	JHS (50)	HS (47)	N	1-3 years (8)	4-6 years (19)	ES 1st-3rd (31)	ES 4th-6th (30)	JHS (38)	HS (32)
Vitamin/Mineral	72	35.0	21.4	27.7	21.5	24.0	42.6	62	62.5	15.8	35.5	43.3	42.1	43.8
Probiotics	55	20.0	39.3	17.0	24.6	18.0	14.9	37	-	36.8	29.0	26.7	26.3	9.4
Growth-promoting	44	5.0	14.3	19.1	20.0	20.0	14.9	6	-	10.5	9.7	3.3	-	-
Protein/amino acid	13	-	-	-	6.2	10.0	8.5	1	-	-	-	3.3	-	-
Academic enhancement	7	-	3.6	2.1	3.1	6.0	-	5	-	5.3	6.5	3.3	2.6	-
Skin repairing	3	-	-	-	-	4.0	2.1	3	-	-	-	-	2.6	6.3
Weight loss	0	-	-	-	-	-	-	1	-	-	-	-	2.6	-
Botanical nutrients/ Aojiru[1]	16	5.0	-	4.3	9.2	10.0	4.3	10	25.0	5.3	-	10.0	5.3	6.3
n-3 PUFA	14	10.0	3.6	6.4	6.2	2.0	6.4	8	-	-	6.5	6.7	-	12.5
Cod liver oil	10	10.0	3.6	6.4	6.2	-	-	5	12.5	10.5	3.2	3.3	-	-
Eye care[2]	6	-	-	2.1	1.5	-	8.5	4	-	-	-	-	7.9	3.1

[1]A powdered drink mix made from green leafy vegetables such as young leaves of *Angelica keiskei* (Miq.) Koidz and Barley, and *Brassica oleracea* L. var. *acephala* DC. [2]Products containing anthocyanin, lutein, and zeaxanthin. Note: Multiple answers.

Table S-3. Types of dietary supplements concomitantly used with asthma medicines. (%)

	Boys (n=226)							Girls (n=141)						
	N	1-3 years (27)	4-6 years (39)	ES 1st-3rd (45)	ES 4th-6th (49)	JHS (35)	HS (31)	N	1-3 years (12)	4-6 years (32)	ES 1st-3rd (25)	ES 4th-6th (21)	JHS (35)	HS (16)
Vitamin/Mineral	62	29.6	23.1	26.7	18.4	25.7	48.4	51	41.7	15.6	40.0	28.6	48.6	50.0
Probiotics	53	11.1	41.0	26.7	28.6	17.1	6.5	33	16.7	31.3	32.0	33.3	17.1	-
Growth-promoting	35	7.4	7.7	15.6	20.4	25.7	12.9	10	-	9.4	16.0	4.8	5.7	-
Protein/amino acid	13	3.7	-	2.2	8.2	11.4	9.7	7	8.3	3.1	12.0	9.5	-	-
Academic enhancement	10	7.4	7.7	2.2	4.1	5.7	-	7	-	6.3	12.0	4.8	2.9	-
Skin repairing	2	-	-	2.2	-	-	3.2	2	-	-	-	-	2.9	6.3
Weight loss	0	-	-	-	-	-	-	0	-	-	-	-	-	-
Botanical nutrients/ Aojiru[1]	12	7.4	-	6.7	6.1	11.4	-	4	8.3	3.1	-	-	2.9	6.3
n-3 PUFA	11	3.7	2.6	6.7	8.2	-	6.5	4	-	3.1	4.0	4.8	-	6.3
Cod liver oil	12	11.1	7.7	4.4	6.1	2.9	-	13	8.3	21.9	12.0	4.8	2.9	-
Eye care[2]	3	-	-	4.4	2.0	-	-	5	-	-	-	4.8	8.6	6.3

[1]A powdered drink mix made from green leafy vegetables such as young leaves of *Angelica keiskei* (Miq.) Koidz and Barley, and *Brassica oleracea* L. var. *acephala* DC. [2]Products containing anthocyanin, lutein, and zeaxanthin. Note: Multiple answers.

Table S-4. Types of dietary supplements concomitantly used with gastrointestinal diseases medicines. (%)

	Boys (n=58)							Girls (n=52)						
	N	1-3 years (12)	4-6 years (10)	ES 1st-3rd (6)	ES 4th-6th (9)	JHS (10)	HS (11)	N	1-3 years (5)	4-6 years (6)	ES 1st-3rd (11)	ES 4th-6th (6)	JHS (10)	HS (14)
Vitamin/Mineral	22	50.0	30.0	50.0	11.1	50.0	36.4	20	20.0	16.7	36.4	16.7	50.0	57.1
Probiotics	11	16.7	30.0	16.7	33.3	20.0	-	5	40.0	16.7	9.1	-	10.0	-
Growth-promoting	3	-	-	-	-	30.0	-	1	-	-	-	16.7	-	-
Protein/amino acid	4	-	-	-	11.1	-	27.3	1	-	-	9.1	-	-	-
Academic enhancement	0	-	-	-	-	-	-	0	-	-	-	-	-	-
Skin repairing	0	-	-	-	-	-	-	0	-	-	-	-	-	-
Weight loss	0	-	-	-	-	-	-	0	-	-	-	-	-	-
Botanical nutrients/ Aojiru[1]	3	-	-	-	22.2	10.0	-	4	-	16.7	18.2	-	10.0	-
n-3 PUFA	3	16.7	-	-	11.1	-	-	4	20.0	16.7	-	-	-	14.3
Cod liver oil	2	-	-	-	11.1	10.0	-	1	-	-	-	-	10.0	-
Eye care[2]	1	-	-	-	-	10.0	-	3	-	-	-	-	20.0	7.1

[1]A powdered drink mix made from green leafy vegetables such as young leaves of *Angelica keiskei* (Miq.) Koidz and Barley, and *Brassica oleracea* L. var. *acephala* DC. [2]Products containing anthocyanin, lutein, and zeaxanthin. Note: Multiple answers.

Table S-5. Types of dietary supplements concomitantly used with psychiatric disorder medicines. (%)

	Boys (n=58)							Girls (n=52)						
	N	1-3 years (0)	4-6 years (1)	ES 1st-3rd (15)	ES 4th-6th (22)	JHS (13)	HS (10)	N	1-3 years (1)	4-6 years (2)	ES 1st-3rd (8)	ES 4th-6th (5)	JHS (10)	HS (20)
Vitamin/Mineral	19	-	-	53.3	22.7	23.1	30.0	20	100.0	50.0	12.5	40.0	40.0	55.0
Probiotics	6	-	100.0	20.0	-	15.4	-	9	-	50.0	50.0	-	20.0	10.0
Growth-promoting	10	-	-	13.3	27.3	7.7	10.0	1	-	-	-	-	10.0	-
Protein/amino acid	4	-	-	-	13.6	7.7	-	0	-	-	-	-	-	-
Academic enhancement	5	-	-	6.7	4.5	23.1	-	0	-	-	-	-	-	-
Skin repairing	0	-	-	-	-	-	-	2	-	-	-	-	-	10.0
Weight loss	0	-	-	-	-	-	-	0	-	-	-	-	-	-
Botanical nutrients/ Aojiru[1]	8	-	-	6.7	9.1	23.1	20.0	1	-	-	-	-	10.0	-
n-3 PUFA	8	-	-	13.3	13.6	23.1	-	2	-	-	-	-	-	10.0
Cod liver oil	0	-	-	-	-	-	-	0	-	-	-	-	-	-
Eye care[2]	2	-	-	-	-	15.4	-	1	-	-	12.5	-	-	-

[1]A powdered drink mix made from green leafy vegetables such as young leaves of *Angelica keiskei* (Miq.) Koidz and Barley, and *Brassica oleracea* L. var. *acephala* DC. [2]Products containing anthocyanin, lutein, and zeaxanthin. Note: Multiple answers.

Table S-6. Types of dietary supplements concomitantly used with antibiotics. (%)

	Boys (n=16)							Girls (n=18)						
	N	1-3 years (1)	4-6 years (1)	ES 1st-3rd (2)	ES 4th-6th (2)	JHS (3)	HS (7)	N	1-3 years (4)	4-6 years (3)	ES 1st-3rd (3)	ES 4th-6th (5)	JHS (2)	HS (1)
Vitamin/Mineral	4	-	-	50.0	50.0	66.7	-	3	25.0	-	33.3	20.0	-	-
Probiotics	2	-	-	-	-	-	28.6	6	75.0	33.3	-	20.0	50.0	-
Growth-promoting	2	-	-	-	-	33.3	14.3	0	-	-	-	-	-	-
Protein/amino acid	2	-	-	-	-	-	28.6	0	-	-	-	-	-	-
Academic enhancement	0	-	-	-	-	-	-	0	-	-	-	-	-	-
Skin repairing	0	-	-	-	-	-	-	0	-	-	-	-	-	-
Weight loss	0	-	-	-	-	-	-	0	-	-	-	-	-	-
Botanical nutrients/ Aojiru[1]	1	-	-	-	50.0	-	-	1	-	-	-	-	50.0	-
n-3 PUFA	1	-	-	-	-	-	14.3	0	-	-	-	-	-	-
Cod liver oil	0	-	-	-	-	-	-	3	-	33.3	66.7	-	-	-
Eye care[2]	0	-	-	-	-	-	-	1	-	-	-	20.0	-	-

[1]A powdered drink mix made from green leafy vegetables such as young leaves of *Angelica keiskei* (Miq.) Koidz and Barley, and *Brassica oleracea* L. var. *acephala* DC. [2]Products containing anthocyanin, lutein, and zeaxanthin. Note: Multiple answers.

Table S-7. Types of dietary supplements concomitantly used with pain killer. (%)

	Boys (n=11)							Girls (n=18)						
	N	1-3 years (0)	4-6 years (1)	ES 1st-3rd (2)	ES 4th-6th (0)	JHS (4)	HS (4)	N	1-3 years (2)	4-6 years (1)	ES 1st-3rd (1)	ES 4th-6th (1)	JHS (4)	HS (9)
Vitamin/Mineral	4	-	-	50.0	-	25.0	50.0	9	50.0	100.0	100.0	-	100.0	22.2
Probiotics	1	-	100.0	-	-	-	-	2	50.0	-	-	100.0	-	-
Growth-promoting	0	-	-	-	-	-	-	0	-	-	-	-	-	-
Protein/amino acid	3	-	-	-	-	25.0	50.0	1	-	-	-	-	-	11.1
Academic enhancement	1	-	-	-	-	25.0	-	1	-	-	-	-	-	11.1
Skin repairing	1	-	-	50.0	-	-	-	1	-	-	-	-	-	11.1
Weight loss	0	-	-	-	-	-	-	0	-	-	-	-	-	-
Botanical nutrients/ Aojiru[1]	0	-	-	-	-	-	-	0	-	-	-	-	-	-
n-3 PUFA	0	-	-	-	-	-	-	0	-	-	-	-	-	-
Cod liver oil	0	-	-	-	-	-	-	0	-	-	-	-	-	-
Eye care[2]	1	-	-	-	-	25.0	-	0	-	-	-	-	-	-

[1]A powdered drink mix made from green leafy vegetables such as young leaves of *Angelica keiskei* (Miq.) Koidz and Barley, and *Brassica oleracea* L. var. *acephala* DC. [2]Products containing anthocyanin, lutein, and zeaxanthin. Note: Multiple answers.

Table S-8. Types of dietary supplements concomitantly used with vitamin/mineral medicines. (%)

	Boys (n=60)							Girls (n=71)						
	N	1-3 years (1)	4-6 years (7)	ES 1st-3rd (7)	ES 4th-6th (8)	JHS (16)	HS (21)	N	1-3 years (4)	4-6 years (5)	ES 1st-3rd (8)	ES 4th-6th (6)	JHS (26)	HS (22)
Vitamin/Mineral	35	-	71.4	57.1	75.0	68.8	42.9	34	75.0	80.0	37.5	50.0	46.2	40.9
Probiotics	2	-	-	14.3	-	-	4.8	4	-	-	25.0	-	7.7	-
Growth-promoting	4	-	-	14.3	-	6.3	9.5	3	-	-	-	16.7	-	9.1
Protein/amino acid	3	-	-	-	12.5	-	9.5	1	-	-	-	-	3.8	-
Academic enhancement	1	-	-	-	12.5	-	-	1	-	-	-	-	3.8	-
Skin repairing	0	-	-	-	-	-	-	0	-	-	-	-	-	-
Weight loss	0	-	-	-	-	-	-	0	-	-	-	-	-	-
Botanical nutrients/ Aojiru[1]	1	-	-	-	-	-	4.8	3	-	-	-	-	3.8	9.1
n-3 PUFA	4	-	-	-	12.5	-	14.3	0	-	-	-	-	-	-
Cod liver oil	2	-	14.3	-	-	6.3	-	2	-	20.0	12.5	-	-	-
Eye care[2]	1	-	-	-	12.5	-	-	4	-	-	-	16.7	11.5	-

[1]A powdered drink mix made from green leafy vegetables such as young leaves of *Angelica keiskei* (Miq.) Koidz and Barley, and *Brassica oleracea* L. var. *acephala* DC. [2]Products containing anthocyanin, lutein, and zeaxanthin. Note: Multiple answers.
